# A peek behind the curtain

**DOI:** 10.1007/s12471-018-1139-8

**Published:** 2018-08-02

**Authors:** M. Kamali-Sadeghian, P. T. G Bot, R. Tukkie, H. J. Wellens, D. J. van Doorn

**Affiliations:** 1 Department of Cardiology, Spaarne Gasthuis, Haarlem, The Netherlands; 2 Department of Cardiology, Maastricht University Medical Center, Maastricht, The Netherlands

## Question

A 67-year-old male was referred to the outpatient clinic of our hospital with crescendo angina pectoris since one month. He had been diagnosed with atrial fibrillation in a general practice three days earlier and treatment with metoprolol and apixaban was initiated. He was an otherwise healthy and active cyclist who smoked a cigar on occasion. There were no other risk factors for coronary artery disease. An echocardiogram performed five years ago because of persistent bradycardia, was normal.

**Fig. 1:**
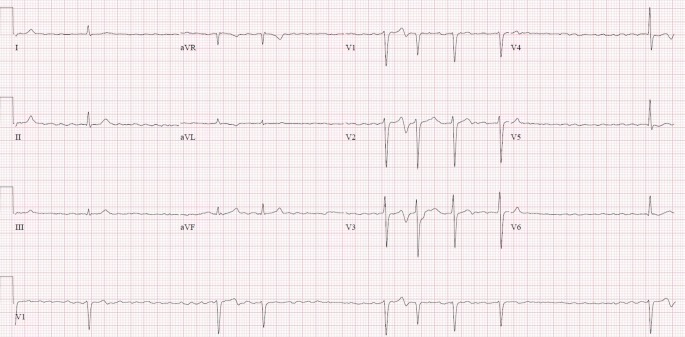
Electrocardiogram on admission in an asymptomatic patient

**Fig. 2:**
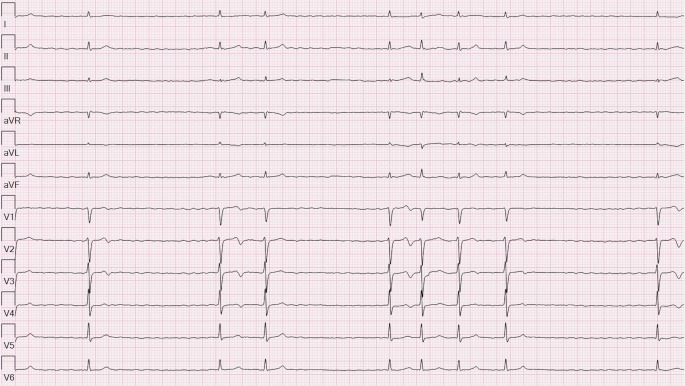
The same electrocardiogram displayed in a 12-lead rhythm strip

Physical and laboratory examination on admission did not reveal any abnormalities. His initial troponin I level was 11 ng/l, with repeat troponin I concentration found at 14 ng/l (normal range 0–10 ng/l). The resting electrocardiogram at presentation is shown in Figs. 1 and 2.

What is your diagnosis based on the electrocardiographic findings?

What would be your next step or treatment?

## Answer

You will find the answer elsewhere in this issue.

